# Challenge data set for macromolecular multi-microcrystallography

**DOI:** 10.1107/S2059798319001426

**Published:** 2019-02-06

**Authors:** James M. Holton

**Affiliations:** a Department of Biochemistry and Biophysics, University of California, San Francisco, CA 94158-2330, USA; b Divison of Molecular Biophysics and Bioengineering, Lawrence Berkeley National Laboratory, Berkeley, CA 94720, USA; c Stanford Synchrotron Radiation Lightsource, SLAC National Accelerator Laboratory, Menlo Park, CA 94025, USA

**Keywords:** protein, simulation, phasing, multi-microcrystallography, radiation damage

## Abstract

A synthetic data set demonstrating a particularly challenging case of indexing ambiguity in the context of radiation damage was generated. This set shall serve as a standard benchmark and reference point for the ongoing development of new methods and new approaches to robust structure solution when single-crystal methods are insufficient. Of the 100 short wedges of data, only the first 36 are currently necessary to solve the structure by ‘cheating’, or using the correct reference structure as a guide. The total wall-clock time and number of crystals required to solve the structure without cheating is proposed as a metric for the efficacy and efficiency of a given multi-crystal automation pipeline.

## Abstract 2

Synthetic macromolecular crystallography diffraction-image data were generated to demonstrate the challenges of combining data from multiple crystals with indexing ambiguity in the context of heavy radiation damage. The nature of the problems encountered using contemporary data-processing programs is summarized.

## 1. Introduction   

Data sets that challenge the capabilities of modern structure-solution procedures, algorithms and software are difficult for developers to obtain for a very simple reason: as soon as a solution is reached, the data set is no longer considered to be challenging. Data sets that are recalcitrant to current approaches are also not available in public databases such as the Protein Data Bank (Berman *et al.*, 2002 ▸) or image repositories (Grabowski *et al.*, 2016 ▸; Morin *et al.*, 2013 ▸) that only contain data used for solved structures. When testing the limits of software, it is generally much more useful to know ahead of time what the correct result will be. This enables the detection and optimization of partially successful solutions at every point in the process, even if downstream procedures fail.

There is a fundamental limit to how small a protein crystal can be and still yield a complete data set (Holton & Frankel, 2010 ▸), so as beams and crystals become smaller and smaller the use of multi-crystal data sets becomes unavoidable. The purpose of the challenge presented here was to represent a situation in which the user decided to take relatively long exposures for each image in order to ensure that the high-resolution spots were visible to the eye. For small crystals, however, much of the useful life of the sample is used up in the first few images using this strategy (Evans *et al.*, 2011 ▸), and the challenge is to reassemble all of the data from a large number of highly incomplete data-collection runs, or wedges.

A low-dose reference data set could greatly reduce the challenges presented here, but only because this is a case of high isomorphism. Real crystals always have some sample-to-sample variability, and may even have more than one crystal habit. Multiple habits are often related by pseudo-symmetry, making it very difficult to distinguish between genuinely heteromorphic crystals and variable indexing software performance. In such cases, which crystal to use as a reference is in no way obvious. Enforcing a presumed unit cell and space group increases the indexing hit rate, but will make the final data worse if intensities are merged from incompatible crystals. For this reason, the present challenge was posed without a reference, and perfect isomorphism was employed only to aid in scoring the results.

## 2. Methods   

### 2.1. Preparation of simulated structure factors (*F*_right_)   

Although it is possible to input *F*_obs_ data into a *MLFSOM* (Holton *et al.*, 2014 ▸) simulation, *F*_obs_ is seldom 100% complete, and any missing *hkl*s provided to *MLFSOM* will be taken as zero when rendering the simulated images, and thus image-processing software will assign them a well measured intensity of zero. This will happen even if the reason for the missing *F*_obs_ was because the spot was saturating the detector in the original experiment, which is a very large and unnatural systematic error. In addition, the anomalous differences of *F*_obs_ are invariably noisy, and are often unavailable. For these reasons, it is convenient to use calculated structure factors, which are always 100% complete, have a well known phase and, by definition, no error in the amplitudes. Additional systematic errors can then be clearly defined and applied, depending on the goals of the simulation.

Calculated structure factors such as those output from refinement programs are typically denoted *F*_calc_, but for clarity here *F*_right_ shall denote the calculated structure factors that are fed into an image simulator. Thus, *F*_right_ denotes the ‘right answer’ used to evaluate the data-processing results. Structure factors obtained from simulated images shall be denoted *F*_sim_, as opposed to *F*_obs_, which will be reserved for actual real-world experimental observations. The distinction is important because the dominant source of systematic error in macromolecular crystallography that leads to the characteristically large ‘*R*-factor gap’ between *F*_obs_ and *F*_calc_ is much larger than all experimental measurement errors combined (Holton *et al.*, 2014 ▸), but the exact nature of this source of error remains unclear. Specifically, refinement against *F*_right_ or *F*_sim_ derived from a simple single-conformer model invariably converges to abnormally low *R*_work_ and *R*_free_ after automated building and refinement. This is a glaring inconsistency with real data, and potentially makes the simulated data unrealistically easy to solve, diminishing their usefulness in benchmarking and debugging. More realistic *R* factors can be obtained by adding random numbers to *F*_right_, but the appropriate random distribution to use is not clear. Instead, values of *F*_right_ were generated here to have a combination of physically plausible systematic errors and one final empirical systematic error.

### 2.2. I1 domain from titin (PDB entry 1g1c): lysozyme’s evil twin   

The titin I1 domain was selected because the PDB entry 1g1c (Mayans *et al.*, 2001 ▸), with unit-cell parameters *a* = 38.3, *b* = 78.6, *c* = 79.6 Å, is the closest non­tetragonal unit cell to that of tetragonal *Gallus gallus* egg lysozyme. The true space group is *P*2_1_2_1_2_1_, and thus represents an excellent challenge to software developers seeking to resolve indexing ambiguity in multi-crystal projects, automatic space-group assignment, detection of non-isomorphism from cell variation (Foadi *et al.*, 2013 ▸) and identification of crystallization contaminants by searching for similar unit cells in a database (McGill *et al.*, 2014 ▸; Simpkin *et al.*, 2018 ▸).

Coordinates and observed structure-factor data for entry 1g1c were downloaded from the PDB (Berman *et al.*, 2002 ▸) and the CIF-formatted structure-factor data were converted to MTZ format using the *CIF*2*MTZ* program from the *CCP*4 suite (Winn, 2003 ▸). The MTZ file header was edited with *MTZUTILS* to make *a* = 38.3 Å and *b* = *c* = 79.1 Å. The deposited coordinates were then refined against the new MTZ file using *phenix.refine* (Adams *et al.*, 2010 ▸) for three macrocycles.

This single-conformer model was used to compute *F*_right_ for a preliminary *MLFSOM* simulation, but downstream analysis suffered from the unrealistically low *R*_free_ < 2% statistics mentioned above. Previous studies (Holton *et al.*, 2014 ▸) found that using *F*_right_ from a multi-conformer model leads to a more realistic *R*_free_, but modern building programs such as *qFit* (van den Bedem *et al.*, 2009 ▸) can easily identify two or three alternate conformations. Real crystals contain trillions of different conformations, but approximating them as a Gaussian distribution simply recovers a canonical *B* factor. Therefore, in order to create physically plausible systematic error that is not easily captured by automated building, twenty alternate conformations were generated for this simulation.

Twenty new PDB files were created from the single-conformer reference by perturbing each atom position, including all waters, with a random coordinate shift consistent with the assigned atomic *B* factor (*B*_atom_) using the *jigglepdb.awk* script distributed with *MLFSOM* (Holton *et al.*, 2014 ▸). Each of the twenty perturbed models was then refined against the re-indexed *F*_obs_ data using *phenix.refine* (Adams *et al.*, 2010 ▸) for ten macrocycles with no free-*R* flags. This operation allowed the coordinates to relax away from any clashes and geometric distortions owing to the unit-cell change and random coordinate shifts and at the same time become more consistent with *F*_obs_. The reason for disabling the free-*R* flags was to avoid creating an artificial *R*_work_ versus *R*_free_ bias in *F*_right_.

The algorithm in the *jigglepdb.awk* program simply shifts each atom along *x*, *y* and *z* using three independent Gaussian deviates taken from a distribution with root-mean-square (r.m.s.) variation equal to (*B*_atom_/24)^1/2^/π. This is the r.m.s. shift that recapitulates the *B* factor at infinite trials. For example, consider a C atom with *B*_atom_ = 5 Å^2^ versus *B*_atom_ = 29 Å^2^. The electron density of both of these cases is readily available using standard crystallography software such as *SFALL* (Winn, 2003 ▸) or *phenix.fmodel* (Adams *et al.*, 2010 ▸), but let us suppose that only *B*_atom_ = 5 Å^2^ is available and we want *B*_atom_ = 29 Å^2^. In that case we must ‘simulate’ an additional *B* factor of 24 Å^2^ by calculating and averaging millions of maps with *B*_atom_ = 5 Å^2^, each after randomly shifting the atom from its starting point. If the r.m.s. shift in any given direction is 0.318 Å, we obtain a map identical to what we would have obtained with *B*_atom_ = 29 Å^2^. This is because an r.m.s. shift of 0.318 Å corresponds to *B* = 24 Å^2^ and *B* factors are additive (5 + 24 = 29). Therefore, atomic shifts of (*B*_atom_/24)^1/2^/π represent the natural deviations that are expected to be found from unit cell to unit cell in the crystal.

The final r.m.s. deviations between these twenty re-refined models ranged from 0.75 to 0.9 Å (0.27–0.34 Å for C^α^ atoms only). Each re-refined model was then edited to change all four methionine S atoms to selenium. The refined solvent parameters *k*_sol_, *B*_sol_, *R*_solv_ and *R*_shrink_ were extracted from each *phenix.refine* run and then used with the selenium-containing coordinates in *phenix.fmodel* to generate twenty complete sets of calculated anomalous structure factors (*F*_model_) out to 1.8 Å resolution. These twenty *F*_model_ sets differed from each other by 14–20%, and were combined together into a single amplitude *F*_r.m.s._ by taking the square root of the mean-square *F*_model_, 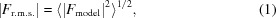
where || denotes the amplitude and 〈〉 the average value. Note that *F*_r.m.s._ is not an error estimate; it is simply an intensity-domain average of the twenty *F*_model_ amplitudes. *F*_r.m.s._ is not equivalent to averaging the electron-density maps (*F*_avg_), which is mathematically identical to averaging *F*_model_ as complex numbers. The difference is that *F*_avg_ assumes that all twenty structures can be found within the coherence length of the beam, whereas *F*_r.m.s._ represents the assumption that the twenty structures make up twenty different types of independently diffracting mosaic domains. The *R* factor between *F*_avg_ and *F*_r.m.s._ was only 3.3%, but since *F*_r.m.s._ represents a physically plausible systematic error, it was carried on to the next step.

An empirical ‘*R*-factor gap’ systematic error was extracted by refining the deposited 1g1c model against the deposited 1g1c data and taking the *F*_obs_ − *F*_calc_ amplitude difference for all observed reflections (*F*_diff_). *F*_diff_ was taken to be an empirical systematic error and added to *F*_r.m.s._ to form *F*_sys_. Reflections missing *F*_obs_ were given *F*_diff_ = 0, and the resulting *R* factor between *F*_r.m.s._ and *F*_sys_ was 18%. Finally, the resolution was made to be slightly better than that available in PDB entry 1g1c with a sharpening filter. This was performed by applying a *B* factor of −15 Å^2^ to *F*_sys_ to form the value of *F*_right_ that was fed into the *MLFSOM* (Holton *et al.*, 2014 ▸) simulation.

### 2.3. Image-simulation runs   

Image simulations were conducted with *MLFSOM* (Holton *et al.*, 2014 ▸) using parameters matching the behavior of an Area Detector Systems (ADSC; Poway, California, USA) model Q315r X-ray detector, which is essentially a powdered Gd_2_O_2_S phosphor bonded to a charge-coupled device (CCD) via a fiber-optic taper (Holton *et al.*, 2012 ▸; Gruner *et al.*, 2002 ▸; Gruner, 1989 ▸; Waterman & Evans, 2010 ▸). These parameters were an electro-optical gain of 7.3 CCD electrons per X-ray photon, an amplifier gain of 4 electrons per pixel intensity unit (ADU), a zero-photon pixel level or ‘ADC offset’ set to 40 ADU, and a readout noise of 16.5 electrons r.m.s. per pixel. An intensity vignette falling to 40% at the edge of each module was used, and the Moffat function for the fiber-coupled CCD point-spread function, as described in Holton *et al.* (2012 ▸), was varied from a *g* value of 30 µm at the center of each module to 60 µm at the corner. The calibration error was set to 3% r.m.s. with a spatial period of 50 pixels. This is in contrast to the true detector behavior of subpixel calibration error (Waterman & Evans, 2010 ▸), but had been found in previous simulations to produce realistic *R*_merge_ values.

Image header values were made to be exact, with the exception of the beam center, which always requires further qualification. The header value was x, y = 154.96, 155.7, which is one pixel off in each direction from the true beam center (155.063, 155.647) in the convention of the *ADXV* diffraction-image viewer program (Szebenyi *et al.*, 1997 ▸; Arvai, 2012 ▸). This one-pixel shift is an example of the unfortunately common array of caveats that can enter into a beam center. Switching between programs that start counting pixels at 1 versus 0 will generate one-pixel shifts, and changing the definition of a pixel location from its center to one of the corners results in half-pixel shifts. More serious changes in beam-center convention involve swapping the *x* and *y* axes, changing the origin among the four corners of the image and two possible mirror flips. Different processing programs have different conventions and, despite significant efforts to standardize them (Parkhurst *et al.*, 2014 ▸), do not always recognize and convert header values properly. The correct values were x_beam 159.353, y_beam 155.063 for *DENZO*/*HKL*-2000 (Otwinowski & Minor, 1997 ▸), BEAM 159.301 155.011 for *MOSFLM* (Leslie & Powell, 2007 ▸), ORGX= 1512.73 ORGY= 1554.57 for *XDS* (Kabsch, 2010 ▸) and origin= −155.063, 159.356, −250 for *cctbx*/*DIALS* (Grosse-Kunstleve *et al.*, 2002 ▸; Winter *et al.*, 2018 ▸). Note that in addition to the *x*–*y* flip between the *ADXV* and *MOSFLM*/*HKL*-2000 conventions, there is a half-pixel difference between the conventions of *MOSFLM* and *HKL*-2000 and a one-pixel difference between the *MOSFLM* and *XDS* conventions. Also, the *XDS* and *DIALS* conventions do not use the beam itself as a reference point, so the values provided above are appropriate only when other program settings declare the detector plane to be perfectly orthogonal to the incident beam. This is usually the case at the start of processing, but refinement of the detector tilt will change these origin values. Detector tilts were simulated but were not included in the image header, specifically 0.365708° forward detector tilt, 0.1145° detector twist and −0.140959° detector rotation about the beam (CCOMEGA), as defined in the *MOSFLM* convention (Leslie & Powell, 2007 ▸), and finally 0.0951363° rotation of the spindle about the vertical axis away from normal to the beam. Although these numbers have many decimal places, they are the exact values that were fed into the simulation.

A total of 100 random orientation matrices with no orientation bias were pre-generated and used to create 100 simulated runs of 15 images each. Each run, or ‘wedge’, began with a new, fresh crystal that was assigned a cube shape with edge dimension selected randomly about a 5 µm average value and 1 µm r.m.s. variation. Crystals larger than 6 µm were cut off by the 6 µm wide square beam. Although misalignment of the crystal with the X-ray beam was not explicitly modeled here, all misalignment does is reduce the illuminated volume, so the variability in crystal size modeled here can equally well be treated as crystal-to-crystal size variation or as same-size crystals with different degrees of misalignment. The only caveat to the latter is that this illuminated volume did not change with rotation, which keeps the ground-truth scale factor simple. The final illuminated volumes are listed in Table 1 ▸.

The X-ray beam was made to have a flux of 1 × 10^12^ photons s^−1^ into a 6 µm wide flat-top profile. The per-image exposure time was 1 s and ΔΦ = 1°. Shutter jitter was set to 2 × 10^−3^ s r.m.s. in the starting and ending Φ values of each image, while beam flicker was taken to be 0.15% Hz^−1/2^ and implemented in ten steps per second. Beam divergence was set to 0.115 × 0.0172° (horizontal × vertical). These are typical measured properties of beamline 8.3.1 at the Advanced Light Source (MacDowell *et al.*, 2004 ▸). Spectral dispersion, however, was set to 0.3% instead of the 0.014% measured from the Si(111) monochromator in order to mimic isotropic unit-cell variations in the sample (Nave, 1998 ▸). The mosaic spread was set to be a uniform disk of sub-crystal orientations with diameter 0.23°.

The X-ray background was also rendered on an absolute scale using realistic thicknesses of the materials in the beam: 20 mm of helium gas between the collimator and beam stop, and 5 µm of liquid water and 4 µm of Paratone-N oil in the beam path. Compton and diffuse scatter from the crystal lattice itself were computed based on the size and the composition of the macromolecule as described in the supplementary materials of Holton *et al.* (2014 ▸). Briefly, at the resolution where the Bragg spots fade into the background this diffuse component of the background converges to the same level as expected from all of the atoms in the protein crystal scattering independently, as if they were a gas.

### 2.4. Simulated radiation-damage model   

**Figure 1:**
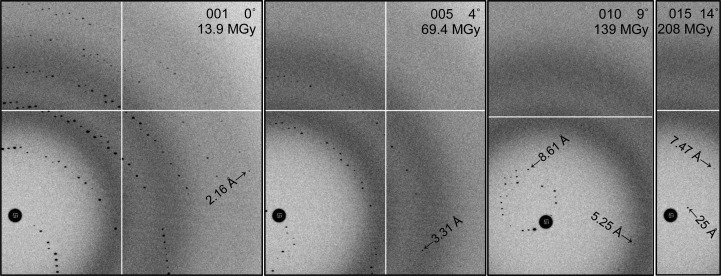
Enlarged sections of diffraction patterns from simulated crystal 016. Six lunes are apparent on image 001, but indexing this wedge still proved problematic. The resolution-dependent exponential fading of spots with dose is exemplified by the rapid loss of high-angle data and the relative persistence of low-angle features. Despite perfect isomorphism, images 004 and higher degraded the overall anomalous signal and images 002 and higher degraded the overall resolution of the final data set.

Radiation damage was simulated in *MLFSOM* (Holton *et al.*, 2014 ▸) with only a simple, resolution-dependent exponential decay of spot intensities with dose using equation (13) from Holton & Frankel (2010 ▸), 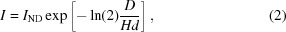
where *I*_ND_ is the intensity that would be observed in the absence of radiation damage, *I* is the spot intensity at dose *D* (MGy), *d* is the resolution of the spot (Å) and *H* is the 10 MGy Å^−1^ resolution dependence of the maximum tolerable dose estimated by Howells *et al.* (2009 ▸). For example, spots in the simulation at 2 Å resolution were made to fade exponentially with dose, reaching half of *I*_ND_ after 20 MGy, and spots at 3.5 Å resolution faded by half at 35 MGy. The dose was calculated assuming that the crystal was bathed in a flat-top beam using the formula 2000 photons µm^−2^ MGy^−1^ from Holton (2009 ▸). This puts the first image at 13.9 MGy (see Fig. 1 ▸), and it should be noted that this end-of-image dose was used for the average dose of the entire image. No attempt was made to average over sub-image decay for this simulation, and the result was that the decay curve appears to be a perfect exponential offset in dose by half an image. Non-isomorphism owing to radiation damage was not simulated, and except for the simple exponential spot fading described above no variation in structure factors or unit cell with dose was employed. In fact, the unit-cell and structure-factor table was identical for all 100 simulated crystals, making this a case of perfect isomorphism. The reason for these unrealistically perfect damage and iso­morphism models was to simplify the estimation of the errors in the cell and damage model introduced by the simulated noise as well as the data-processing algorithms themselves.

It is noteworthy that although (2) is consistent with 13 distinct studies of crystals and single particles using both X-rays and electrons surveyed by Howells *et al.* (2009 ▸) over a resolution range of 2–600 Å, it is not equivalent to a *B* factor that increases with dose. This is incongruous with popular scaling programs, which use a quadratic (*B* factor) rather than a linear (2) resolution dependence for spot fading (Blake & Phillips, 1962 ▸; Evans, 2006 ▸). Borek *et al.* (2013 ▸) describe one exception using *SCALEPACK*, but this non-Gaussian scaling option was only tested at low doses and is not the default. This damage model is therefore an example of a systematic error between the simulation and the internal models of scaling programs. These differences are detailed in Section 3.3, but it should be noted that the systematic error between reality and either of these decay models is no doubt even more complex. In this work, the average trend of spot fading versus resolution was used as the sole manifestation of radiation damage.

## 3. Results and discussion   

In order to demonstrate the utility of this challenge, some discussion of the difficulties encountered when trying to solve the structure using *MOSFLM* (Leslie & Powell, 2007 ▸), *LABELIT* (Sauter & Poon, 2010 ▸), *HKL*-2000 (Otwinowski & Minor, 1997 ▸), *XDS*/*XSCALE* (Kabsch, 2010 ▸), *DIALS* (Winter *et al.*, 2018 ▸), *PHENIX* (Adams *et al.*, 2010 ▸), the *CCP*4 suite (Winn, 2003 ▸) and *BLEND* (Foadi *et al.*, 2013 ▸) is provided here. Specific bugs and program-to-program differences will not be detailed here as software is continuously improving and contemporary shortcomings have little archival value, but the algorithmic challenge of simultaneous speed and robustness will be evaluated. The performance of particular programs with this data set is best described by their authors, such as Gildea & Winter (2018 ▸).

### 3.1. Automatic indexing   

Despite the high degree of similarity between these 100 simulated crystals, automated indexing was not always successful. Depending on the software used, the choice of images and the settings for spot picking and cell restraints, failures ranged from exiting with an error message to confidently arriving at an incorrect Niggli cell, usually with one or more of the primitive cell dimensions doubled. This type of mis-indexing could not be corrected by downstream re-indexing programs such as *POINTLESS* (Evans, 2006 ▸, 2011 ▸), and thus represents a significant barrier to including these particular wedges.

**Figure 2:**
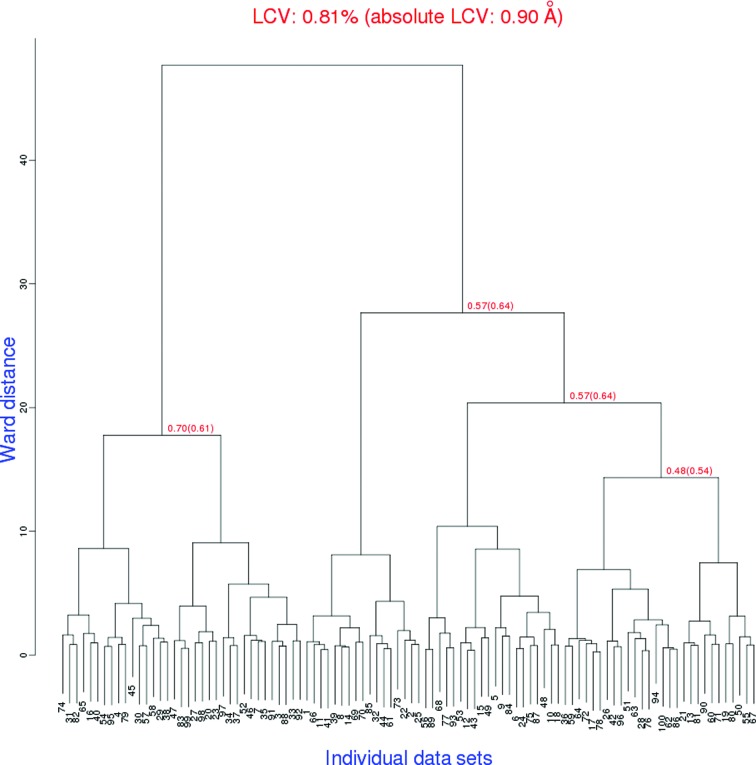
*BLEND* (Foadi *et al.*, 2013 ▸) dendrogram of unit cells obtained from *XDS* (Kabsch, 2010 ▸) processing. Although the clustering suggests groups of related crystals, the true underlying unit cells and structure factors were identical for all 100 wedges. The unit-cell variation shown here is therefore entirely owing to the impact of random noise on indexing and cell refinement.

A naïve user might even mistake such mis-indexing for evidence of variations in crystal habit, so it is important to note here that there was no difference in quality between any of these simulated crystals. All wedges had the same resolution and the same decay rate and were perfectly isomorphous. The true unit cells were all identical as well, which allowed calibration of the influence of random noise on cell refinement. Clustering the refined unit cells using *BLEND* (Foadi *et al.*, 2013 ▸) demonstrated that an LCV of ∼1% does not necessarily imply non-isomorphism, and that even random relationships still produce a dendrogram with major and minor branching (Fig. 2 ▸).

Aside from orientation, the only major difference between the simulated crystals was the illuminated volume, which varied over a factor of 24 (Table 1 ▸). However, neither the smallest (037) nor the largest (092) simulated crystal had indexing problems. The most problematic crystals were 016, 064, 065, 086 and 095, all of which have one reciprocal-cell axis close to parallel to the incident beam. This situation can cause problems in indexing because the information about the cell axis near the beam is maximally distorted by the Ewald sphere and may even be missing entirely if the crystal diffracts poorly and produces only one lune (Brewster *et al.*, 2018 ▸). However, all of these problematic wedges diffracted to 1.8 Å resolution and displayed 3–6 clear lunes, so the reason for these failures is not immediately clear. In addition to these five problem crystals, four others, 051, 054, 062 and 063, failed with most combinations of images but not all, and 11 more, 004, 006, 010, 019, 065, 068, 086, 094, 097 and 098, usually succeeded but failed with at least one combination of images. Since the major difference was the crystal orientation, the indexing algorithm itself may be considered to be a source of orientational bias in multi-crystal data, even if the true orientation distribution is isotropic.

In general the fastest programs had the highest failure rates, whereas more complex algorithms took longer but arrived at the correct Niggli cell more reliably, such as that of Sauter & Zwart (2009 ▸). Execution times varied from 0.3 to 9 s across the programs tested, so the tradeoff between speed and robustness is significant. However, these same more complex algorithms were vulnerable to other considerations, such as weak images. For example, *LABELIT* indexing with images 1 and 15 failed in 78/100 cases, but the same program given images 1 and 4 found the correct lattice for 100/100 cases. A combinatorial approach scanning over image selection and other program settings would no doubt be most robust, but would also consume the most computing resources.

Automatic space-group determination also had its flaws. Essentially all indexing software tested arrived at a tetragonal solution, which is not intrinsically problematic until after the merging step, but the completeness of any given single wedge was so low (∼10%) that few symmetry operators could be eliminated for any particular wedge taken in isolation. For example, *POINTLESS* (Evans, 2006 ▸, 2011 ▸) assigned most of the 100 simulated crystals to space groups *P*1 (35%) or *P*2 (23%), while some were assigned to *P*222 (11%), *C*2 (12%) or *P*422 (9%) and in rare cases to *C*222 or *P*4, indicating that the true space group is not obvious from the primary data. It is commonplace to assign the highest symmetry possible during processing in order to maximize the completeness of each wedge and therefore the overlap with other wedges to make cross-crystal scaling simpler and more robust. However, pursuing this strategy invariably ended with what appeared to be extremely noisy data that did not merge well and appeared to be twinned. The final *R* factor between *F*_sim_ and *F*_right_ was 53%. The most robust strategy and unfortunately the most computationally intensive remained independently pursuing processing, scaling, merging and combining data in all possible point groups separately, and in addition scanning over all possible radiation-damage cutoffs. This is a large number of combinations, but the correct point group (222) and cutoff (three images) were only clear when both were applied at the same time.

One trick that proved to be helpful in solving this data set (Diederichs, 2016 ▸; Gildea & Winter, 2018 ▸) is to initially drop all symmetry to *P*1. This avoids overestimation of symmetry and worked well for the present challenge data. However, it is expected that for real-world cases that have poorer resolution and more incomplete wedges working in *P*1 will be limiting. For example, cell refinement is less stable when the lattice is completely unrestrained. The connectivity between wedges is also minimized by comparing them in *P*1 because many observations that would be symmetry-equivalent in the true crystal symmetry are not equivalent in *P*1. This lack of overlap makes resolving the indexing ambiguity harder or even impossible in the limit of sparse data from few crystals. It is expected that finding a way to reliably identify and take advantage of the internal symmetry within each wedge will be a valuable future development.

### 3.2. Cheating   

In order to demonstrate an ideal solution to this challenge, the simulated data were processed using *F*_right_ as a reference for the unit cell and structure factors. This eliminated any indexing ambiguity. The unit cell and space group were also fixed to the correct values during indexing, refinement and integration in *MOSFLM* (Leslie & Powell, 2007 ▸). The best radiation-damage cutoff was determined empirically by scaling and merging all 100 correctly indexed wedges together with *POINTLESS*/*AIMLESS* (Evans, 2011 ▸) and comparing the final merged structure factors with *F*_right_.

**Figure 3:**
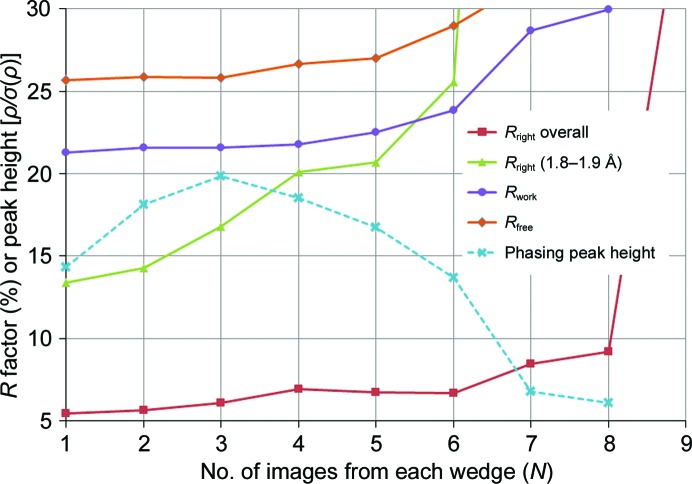
Graph of the relative error (*R*_right_) between the correct structure factor (*F*_right_) and the structure factor obtained from scaling and merging the first *N* images from all 100 simulated crystals (*F*_sim_). Also shown are *R*_work_ and *R*_free_ from refinement to convergence of the correct starting model against *F*_sim_ from *N*-image data. Despite perfect isomorphism, fewer images resulted in better agreement. The *y* axis also represents the maximum peak height found in the phased anomalous difference Fourier (dashed line). Phases were obtained by removing all Se atoms before refining to convergence against *F*_sim_. The phasing signal is maximized at *N* = 3.

The optimum cutoff to optimize weak, high-resolution data was to use only the first image, as shown in Fig. 3 ▸. Although scaling programs such as *AIMLESS* take a ‘run’ of images, for this case each run started and ended with image ‘1’, a strategy that also eliminates all partially recorded reflections. Using just the first image from each wedge also minimized the overall *R*_work_ to 21.3% and *R*_free_ to 25.7% after refining the selenated reference model PDB entry 1g1c to convergence with *REFMAC* (Murshudov *et al.*, 2011 ▸). This is most likely because the increase in *R*_right_ with increasing *N* shown in Fig. 3 ▸ was due to unstable scaling. After correcting for the known crystal volumes (Table 1 ▸), the r.m.s. variation in the scale factor assigned to spots in the 1.8–1.9 Å bin was 18% for *N* = 5 but was only 1.4% at *N* = 1. This was almost entirely owing to variation in the scaling *B* factor, which was actually invariant from crystal to crystal in the simulation. The reason for this instability is suspected to be the incongruence of radiation-damage models detailed in Section 3.3.

The optimum anomalous signal was attained using the first three images of each wedge (Fig. 3 ▸), and structure solution was straightforward using automated phasing pipelines, much as reported by Gildea & Winter (2018 ▸). Structure solution was also possible with fewer data, down to crystals 001–042, with *SHELXC*/*D*/*E* (Sheldrick, 2015 ▸; Usón & Sheldrick, 2018 ▸), indicating the threshold of solvability with ideal data processing. All four correct selenium sites, as evaluated with *phenix.emma*, were found with *SHELXD* using as few data as crystals 001–029 with CC_all_/CC_weak_ at 30/20%. Applying a further cheat of providing *SHELXE* with the correct selenium and sulfur sites allowed the application of the twofold NCS, making structure solution possible down to crystals 001–036. Better results are expected with further cheats, such as directly correcting the exponential spot decay, but this was not attempted in the present work. Nondefault parameters that were necessary for success were instructing *SHELXD* to find four sites with a resolution cutoff of 3.5 Å and MIND -3.5. For *SHELXE* using the correct sites the required options were -s0.53 -n2 -a100 -w0.3 -F0.7 -t5 -L1 -B3. Using the *SHELXD* sites, solution was possible down to crystals 001–040 with the options -s0.53 -a100 -t1 -B3 -L1. No parameters could be found to solve the structure using crystals 001–035, despite a systematic search over >9000 distinct sets.

A script provided as supporting information reproduces the solutions described above, but it should be noted that near the threshold any protocol will be fragile. Changing any parameter, such as using a processing program other than *MOSFLM*, or even using different CPU types, could make or break the solution. As crystallographic software evolves these sensitivities are expected to disappear and perhaps new ones will manifest. It is therefore recommended to start with the robust case of merging 100 crystals and then to start dropping crystals from the tail end until the limitation of the pipeline of interest is found. It is at this threshold that the vulnerabilities of any given algorithm are most easily detected and corrected.

### 3.3. Resolution dependence of radiation damage   

The non-Gaussian nature of the damage model used in this simulation was unexpectedly detrimental to contemporary scaling procedures, so here we shall place this empirical decay equation into context with the conventional scale-and-*B*-factor model. It is instructive to recast (2) in the same form as a *B* factor [exp(−*Bs* ^2^)] by defining *A* = ln(2)*D*/*H*, substituting the resolution *d* with the reciprocal scattering-vector length *s* = (2*d*)^−1^ and converting intensities (*I*) to structure factors (*F*) by taking the square root of both sides. The factor of two in the switch from *d* to *s* is canceled by the switch from intensities to structure factors, and we arrive at
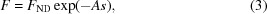
where *F*_ND_ is the structure factor of the damage-free unit cell. This rearranged spot-fading formula immediately suggests a Taylor expansion in the exponent, demonstrating the relationship between *A* and *B*, and perhaps additional factors such as *C*. Let us briefly entertain this formalism, and write
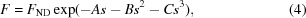
where *B* is the usual *B* factor (8π^2^〈*u*_*x*_^2^〉), in which *u_x_* is the component of the Gaussian-distributed atomic displacement vector **u** in the direction normal to the Bragg plane and 〈〉 denotes the mean over all atoms. Similarly, *A* = 2π*w*_fhm_, where *w*_fhm_ is the full-width at half-maximum of atomic displacements taken from the multivariate Cauchy–Lorentz distribution, 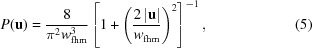
where *P*(**u**) is the normalized probability of atomic displacement vector **u** and || denotes the vector magnitude (in Å). This distribution resembles a Gaussian but has heavier tails, indicating a much higher ratio of large-scale to small-scale movements than would be expected from a Gaussian distribution. Generating this distribution must be performed with care because one cannot simply apply three independent displacements along *x*, *y* and *z*, as this creates a highly anisotropic three-dimensional histogram. Rather, a random direction for **u** must first be chosen and (5) applied along its axis.

It was argued by Debye (1914 ▸) that all terms except *Bs* ^2^ in (4) vanish when averaged over the large number of atoms in the crystal (equation I.26 in James, 1962 ▸), but this is only the case when the distribution of atomic displacements converges to a Gaussian via the central limit theorem. There are random distributions that do not obey the central limit theorem, and the Cauchy–Lorentz distribution is one example. In fact, combinations of Cauchy–Lorentz deviates always converge to another Cauchy–Lorentz distribution, forming an analogous but distinct version of the central limit theorem.

Strictly speaking, the falloff of intensity with resolution owing to any distribution of atomic displacements is the Fourier transform of that distribution. The Fourier transform of a Gaussian atomic displacement distribution is another Gaussian (the *B* factor), and the Fourier transform of a Cauchy–Lorentz distribution is an exponential in reciprocal space, as in (3). If the manifestation of radiation damage is a *B* factor that increases linearly with dose, then the spot-fading half-dose would be related to the square of resolution, not linearly. The observation by Howells of a linear relationship between resolution and spot-fading half-dose therefore implies a direct proportionality between dose and the width of the distribution of atomic displacements, 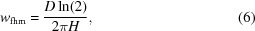
where *D* is the dose in MGy, ln(2) is the natural log of 2 and *H* is the 10 MGy Å^−1^ trend observed by Howells. Here, we use the full-width at half-maximum to describe the Cauchy–Lorentz histogram rather than the r.m.s. variation because the r.m.s. variation of a Cauchy–Lorentz distribution is undefined, as is its mean. A physically reasonable explanation for the departure from Gaussian-distributed atomic displacements may be that large enough displacements require neighboring atoms to move out of the way, creating additional large **u** vectors of similar magnitude and direction, and leading to a higher than ‘normally’ expected population of large **u** vectors. Cracking and slipping of lattice fragments relative to each other may be examples of such concerted movements.

As a historical aside, the appearance of the letter *B* as the second term in (4) invites speculation that it is the origin for the choice of the letter *B* to indicate the Debye–Waller–Ott factor, and therefore a natural place for *A* and *C* factors. This is not actually the case. The first use of *B* to describe Debye’s disorder parameter appeared in Bragg (1914 ▸), and therein the letter *A* was used to encapsulate the overall scale factor, which is in no way analogous to the Cauchy–Lorentz term in (4). What is more, the *C* factor does not relate to any physically reasonable distribution because its corresponding real-space displacement histogram has negative population values, and probabilities cannot be negative. So, although (4) resembles a Taylor expansion in the exponent, only the first two terms *A* and *B* correspond to physically plausible distributions.

## 4. Conclusions   

The challenges to macromolecular structure determination using data from a large number of small crystals lie primarily in the combinatorial nature of the data analysis. Recent landmark achievements such as those reported by Brehm & Diederichs (2014 ▸), Liu & Spence (2014 ▸), Gildea & Winter (2018 ▸), Diederichs (2016 ▸, 2017 ▸) and, in this issue, Foos *et al.* (2019 ▸) represent important mathematical advances in handing this problem and significant practical progress towards solving the present challenge. The indexing-ambiguity problem itself may now be regarded as solved, with the proviso that current approaches are still vulnerable to incorrect lattice assignment, such as cell doubling, and radiation-damage cutoffs during processing. These choices are still up to the user, and since the correct choice is generally not clear until the structure has been solved, the only robust strategy remains an exhaustive evaluation of all possible lattice-type and damage-cutoff options. By ‘cheating’ this work was able to solve the challenge structure using only the first 36 crystals of the 100 presented, and further work that can approach or surpass this number without cheating will directly translate to real-world projects finishing earlier and using fewer difficult-to-produce isomorphous crystalline samples.

It is tempting to suggest overcoming indexing problems by using a pair of orthogonal alignment shots prior to data collection, but since only the first three images appear to be useful before the data quality degrades this strategy is not recommended. Lowering the exposure time and covering more of reciprocal space with the same dose is expected to improve the indexing performance, but this strategy is not applicable to the problem of serial crystallography (Wiedorn *et al.*, 2018 ▸; Chapman *et al.*, 2011 ▸), where particularly at XFEL sources only one image is available from each sample. The limit of how weak individual images can be before resolution begins to degrade will be the subject of a future challenge, but recent results have shown that this limit can be quite low (Lan *et al.*, 2018 ▸; Parkhurst *et al.*, 2016 ▸). It is further expected that as radiation-damage processes become better understood and correctable including more images will improve data quality rather than degrade it.

The challenge proposed here is to beat the 36-crystal limit and solve this structure by anomalous phasing without ‘cheating’ in any way. In the real world a reference data set may not be available or appropriate if the crystals are not very reproducible. Realistic solutions to the indexing ambiguity must also be able to handle the inaccurate first-pass symmetry determination that is inherent to highly incomplete data sets, and automatic radiation-damage cutoffs must become more reliable to be of practical use.

## Supplementary Material

Shell script for reproducing the `cheat' solution to the challenge.. DOI: 10.1107/S2059798319001426/ba5297sup1.exe

Challenge data set for macromolecular multi-microcrystallography URL: https://doi.org/10.18430/microfocus_challenge_2011

## Figures and Tables

**Table 1:** Simulated crystal volumes (µm^3^) The true scale factor of the spots from each simulated data set is directly proportional to the simulated crystal volume, which was chosen randomly for each crystal. The actual values used in the simulation are listed here and may be used to check the accuracy of scaling programs as in Section 3.2 because no other variables such as the X-ray beam flux or even the structure factors were varied from crystal to crystal. The only remaining correction after this is the resolution-dependent scale factor of the simulated radiation damage described in Section 3.3.

Crystal	Volume	Crystal	Volume	Crystal	Volume	Crystal	Volume	Crystal	Volume
001	225	021	139	041	132	061	50.3	081	105
002	56.3	022	232	042	234	062	99.5	082	230
003	63.9	023	155	043	46.9	063	196	083	171
004	220	024	114	044	75.9	064	102	084	122
005	186	025	38.4	045	51.6	065	229	085	56.8
006	89.2	026	155	046	89.1	066	161	086	90.5
007	52.2	027	46.7	047	230	067	72.4	087	90.2
008	249	028	60.7	048	56.7	068	14.5	088	171
009	185	029	70.7	049	97.8	069	131	089	186
010	110	030	166	050	153	070	37.5	090	128
011	166	031	143	051	237	071	207	091	42.2
012	121	032	132	052	87.4	072	159	092	295
013	160	033	213	053	130	073	88.4	093	240
014	60.4	034	27.8	054	128	074	60.2	094	148
015	189	035	210	055	86.4	075	190	095	51.5
016	39.4	036	100	056	127	076	39.2	096	134
017	47.6	037	12.5	057	52.8	077	186	097	46.3
018	123	038	228	058	104	078	78.5	098	15.8
019	277	039	210	059	146	079	108	099	201
020	71.4	040	83.7	060	102	080	31.2	100	111
